# A Literature Review of the Management of Nasal Polyps: Biologics Combined With Endoscopic Surgery

**DOI:** 10.7759/cureus.93657

**Published:** 2025-10-01

**Authors:** Manuel E Castillo Cortorreal, Ana G Martínez Areché, Samuel Román Ledesma, Rosa Ileana De los Santos Estevez, Alfida Luisa Fernandez Rodriguez, Greisha Marie Quiles Robles, Obinna Eleazar, Andreina Rosario Rosario

**Affiliations:** 1 Otorhinolaryngology, Universidade Castelo Branco, Río de Janeiro, BRA; 2 Plastic Surgery, Hospital Casa do Portugal, Río de Janeiro, BRA; 3 Medicine, Autonomous University of Santo Domingo (UASD), Santo Domingo, DOM; 4 Medicine, Universidad Iberoamericana (UNIBE), Santo Domingo, DOM

**Keywords:** ahora no, chronic rhinosinusitis, nasal polyps, otorrino, si

## Abstract

Chronic rhinosinusitis with nasal polyps (CRSwNP) is a multifactorial inflammatory condition commonly linked to other respiratory disorders. Symptoms include nasal obstruction, loss of smell, and facial pressure. Treatment strategies include medical therapy, primarily intranasal corticosteroids and biologics, as well as surgical approaches such as functional endoscopic sinus surgery (FESS). This review compares their efficacy, safety, and long-term outcomes to guide evidence-based management.* *A narrative literature review was conducted in accordance with the Scale for the Assessment of Narrative Review Articles (SANRA) guidelines. Databases searched (2020-2025) included PubMed, Scopus, Cochrane, and Embase. Inclusion criteria: adult patients with bilateral nasal polyposis comparing biologics versus endoscopic surgery. Excluded were pediatric studies, neoplastic polyps, or non-comparative reports. Twenty-five studies met the inclusion criteria. Data extracted included interventions, follow-up, outcomes (SNOT-22, olfaction, recurrence), and sample sizes. A third reviewer resolved discrepancies.* *From 1,520 initial records, 25 studies were selected for inclusion. Findings showed that both FESS and biologics significantly improve SNOT-22 scores and symptom relief. FESS provided faster reduction in polyp burden, while biologics (e.g., dupilumab) offered better olfactory outcomes and reduced corticosteroid use. Recurrence rates post-FESS ranged from 15% to 22%. Sequential strategies (surgery followed by biologics) demonstrated superior long-term control. Cost-effectiveness favored surgery in the short term, while biologics became viable over three years in cases of severe, refractory disease. Both FESS and biologics are effective for CRSwNP, with biologics offering superior olfaction restoration and steroid reduction. Surgery remains essential for rapid relief and anatomical correction. A personalized, multimodal approach based on disease severity, patient comorbidities, and treatment response ensures optimal outcomes. Wider access to biologics and further real-world trials will refine long-term treatment strategies.

## Introduction and background

Nasal polyposis is a clinical entity within the broader spectrum of chronic rhinosinusitis with nasal polyps (CRSwNP), characterized by persistent inflammation of the nasal and paranasal mucosa accompanied by the development of benign polypoid masses [1]. This condition, which arises from a multifactorial etiology, affects approximately 1-4% of the global population. It is more commonly seen in individuals over 40 years of age and is frequently associated with comorbidities such as asthma, aspirin-exacerbated respiratory disease, and cystic fibrosis [2].

Histologically, nasal polyps are composed of edematous stroma infiltrated by eosinophils, neutrophils, and lymphocytes, alongside glandular hyperplasia and increased vascular permeability. Unlike other forms of sinus inflammation, CRSwNP is driven by a Th2-skewed immune response, characterized by elevated levels of cytokines such as IL-4, IL-5, and IL-13, which promote eosinophilia and local IgE production [3].

Clinically, patients typically report bilateral nasal obstruction, hyposmia or anosmia, anterior or posterior rhinorrhea, and facial pressure, all of which can significantly impact quality of life. Diagnosis typically involves nasal endoscopy or anterior rhinoscopy, often complemented by CT scans of the paranasal sinuses. The Lund-Mackay scoring system is often used to assess disease extent [4].

The optimal management of nasal polyps has long been a subject of debate in otolaryngology, owing to the condition’s chronic, relapsing nature, its multifactorial etiology, and its substantial impact on patient quality of life. One of the key challenges lies in defining treatment pathways when multiple effective modalities, ranging from corticosteroids and biologics to surgical interventions, are available, each with distinct benefits, limitations, and economic implications [1,2,5].

Over recent decades, the management of nasal polyps has evolved, focusing on controlling underlying chronic inflammation, preventing recurrence, and restoring nasal function. Currently, treatment such as EPOS 2020 emphasizes individualized care, underscoring that while both surgery and biologics are effective, patient phenotype, comorbidities, and healthcare system resources must guide decision-making [5].

Over recent decades, the management of nasal polyps has evolved, focusing on controlling underlying chronic inflammation, preventing recurrence, and restoring nasal function. Currently, treatment options are broadly categorized into medical and surgical approaches. Medical management centers around intranasal corticosteroids as the first-line therapy, supplemented in some cases by antihistamines, saline irrigations, specific immunotherapy, and, more recently, biologic agents targeting immune pathways such as IL-4 receptor antagonists (dupilumab), anti-IgE (omalizumab), and IL-5 inhibitors (mepolizumab, benralizumab) [6,7].

Surgical intervention, particularly functional endoscopic sinus surgery (FESS), is typically reserved for patients with persistent or recurrent disease despite optimal medical therapy, which, as defined by EPOS 2020, consists of daily intranasal corticosteroids (e.g., mometasone, budesonide, fluticasone) combined with saline irrigations for at least 8-12 weeks, supplemented by short courses of systemic corticosteroids during severe exacerbations. Adjunctive therapies, such as antihistamines or leukotriene receptor antagonists, may be used in selected cases. Patients with severe, recurrent, or steroid-dependent disease may benefit from biologic therapies (e.g., dupilumab, mepolizumab, omalizumab), with treatment response assessed at 16-24 weeks. First described by Messerklinger and later popularized by Stammberger, FESS enables the targeted removal of polyps, improves sinus ventilation, and restores nasal anatomy [8]. Numerous studies have demonstrated that FESS significantly improves symptoms, reduces the need for systemic corticosteroids, and enhances the efficacy of topical treatments. Beyond symptom control and anatomical correction, FESS may also enhance the efficacy of ongoing medical therapy by facilitating improved penetration of topical corticosteroids and saline irrigations into the sinus mucosa [9]. However, surgery carries inherent risks, including bleeding, synechiae formation, orbital injury, and cerebrospinal fluid leaks. As such, careful patient selection is critical [10].

In recent years, the therapeutic landscape has expanded with the advent of biologic therapies. Monoclonal agents, such as dupilumab, have revolutionized treatment paradigms by providing disease control without the need for repeated systemic corticosteroid use or multiple surgical interventions. Trials such as LIBERTY NP SINUS-24 and SINUS-52 have demonstrated significant reductions in polyp size, improvements in SNOT-22 scores (Sino-Nasal Outcome Test), and restoration of olfactory function [11].

Nonetheless, access to biologics remains limited in many regions due to high costs and strict eligibility criteria [12]. This reality has reignited discussions surrounding the optimal timing and selection of surgical versus long-term medical treatment strategies, especially within healthcare systems with constrained resources.

Despite an abundance of literature, several controversies persist: the ideal timing for surgical intervention, the long-term safety of systemic corticosteroid use, and the definitive role of biologics as a first-line option. Some authors advocate for an initial surgical approach followed by maintenance medical therapy as the most effective strategy, while others favor a stepwise model beginning with conservative medical management [13].

Given these ongoing debates, a critical and systematic review of the current literature is essential. This review aims to compare the efficacy, safety, and long-term outcomes of both medical and surgical treatment modalities for nasal polyposis. The goal is to provide a comprehensive, evidence-based analysis that informs future clinical practice guidelines and optimizes patient outcomes.

## Review

Study design

A critical narrative review of the scientific literature was conducted, focusing on studies that compared medical and surgical treatments in adult patients with a confirmed diagnosis of bilateral nasal polyposis, as established through both clinical and radiological evaluations. The review adhered to the methodological rigor and transparency principles recommended by the Scale for the Assessment of Narrative Review Articles (SANRA) guidelines.

Search strategy

The literature search was carried out between February and May 2025 across the following databases: PubMed/MEDLINE, Cochrane Library, Scopus, and Embase. Boolean search combinations used were (“nasal polyps” OR “chronic rhinosinusitis with nasal polyps” OR “CRSwNP”) AND (“medical therapy” OR “biological therapy” OR “intranasal corticosteroids”) AND (“surgical treatment” OR “FESS” OR “functional endoscopic sinus surgery”) AND (“comparative studies” OR “outcomes” OR “management”).

The results were limited to articles published in English or Spanish between January 2020 and April 2025. Priority was given to cohort studies, randomized controlled trials (RCTs), systematic reviews, and meta-analyses.

Inclusion criteria were adult patients (≥18 years) with a confirmed diagnosis of nasal polyposis and studies directly comparing medical and surgical treatments that reported clinical outcomes such as SNOT-22 scores, polyp size reduction, olfactory function, quality of life, recurrence rates, need for revision surgery, and a minimum follow-up of six months. Exclusion criteria included studies that focused on unilateral polyps or those associated with neoplastic processes. These studies focused on pediatric populations or lacked comparative data, individual case reports, letters to the editor, editorials without original data, and studies without full-text availability.

Two independent reviewers (R1 and R2) screened studies by reviewing titles and abstracts. In cases of disagreement, a third reviewer was consulted. To mitigate potential bias, data extraction was performed independently and in duplicate, with discrepancies resolved through discussion or adjudication by the third reviewer. Additionally, a standardized data extraction form was used to ensure consistency across studies. Extracted data included year of publication, country of origin, study design, sample size, interventions compared, outcome measurement tools, duration of follow-up, and key clinical findings.

**Figure 1:**
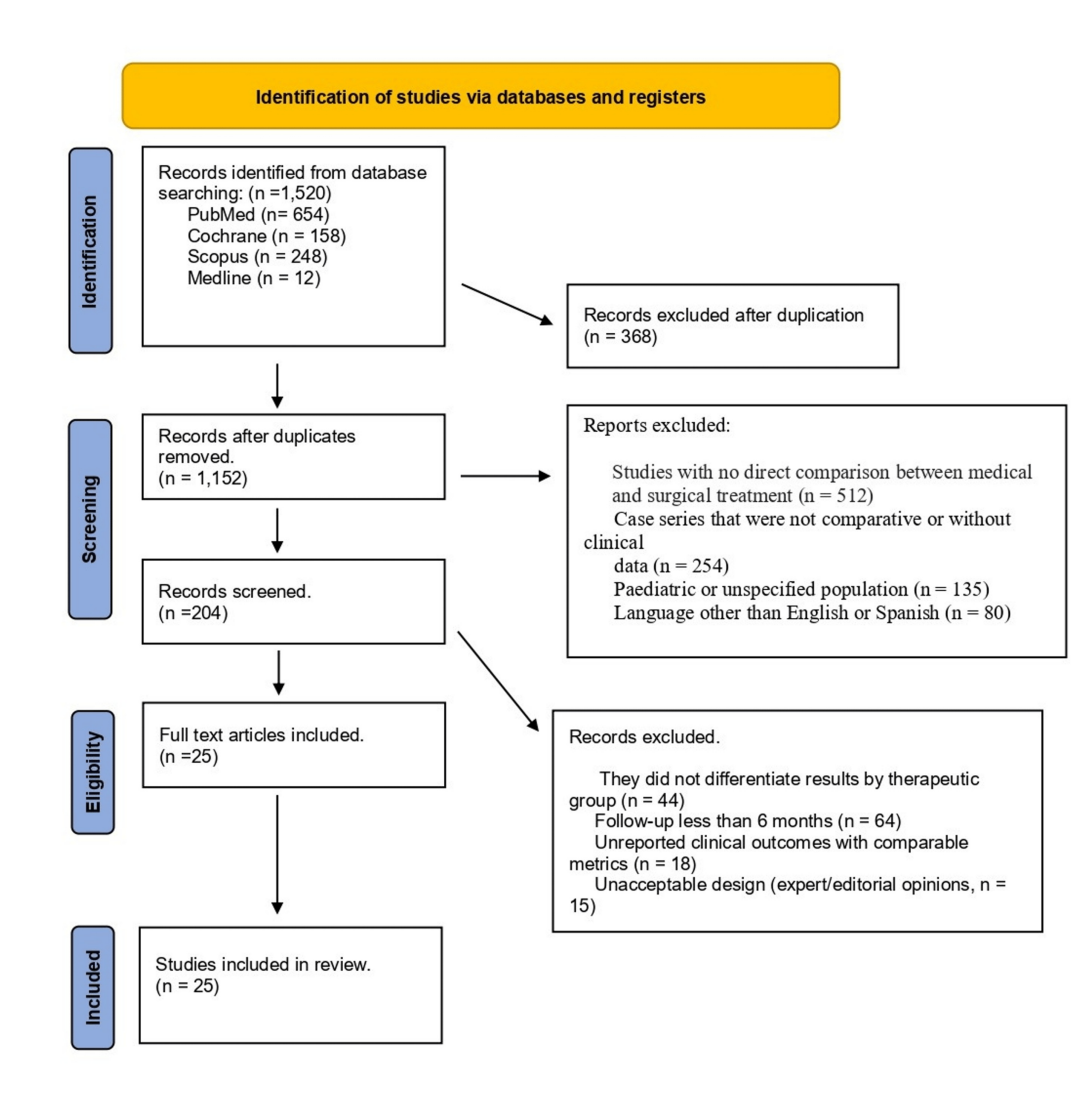
PRISMA flow diagram PRISMA: Preferred Reporting Items for Systematic Reviews and Meta-Analyses

A total of 1,520 records were identified using the PubMed, Embase, Cochrane, and Scopus databases. After removing duplicates (n = 368), 1,152 titles and abstracts were evaluated. A total of 948 were discarded because they did not meet a design criterion. Two hundred four were excluded because they were clinical cases, reviews without original data, pediatric samples, or did not include direct comparisons. Ultimately, 25 studies were included in the qualitative analysis (Figure 1, Table 1).

**Table 1:** Comparative summary of clinical studies on CRSwNP RCT: randomized controlled trial, ESS: endoscopic sinus surgery, FESS: functional endoscopic sinus surgery, INCS: intranasal corticosteroids, APPS: awake patient polyp surgery, PNN: posterior nasal nerve, NPS: nasal polyp score, NC: nasal congestion, SNOT-22: 22-item Sinonasal Outcome Test, QoL: quality of life, QALY: quality-adjusted life year, SAQ: Sino-Nasal Assessment Questionnaire, VAS: Visual Analog Scale, CT: computed tomography, NSAID-ERD: nonsteroidal anti-inflammatory drug-exacerbated respiratory disease, CRSwNP: chronic rhinosinusitis with nasal polyps, ECRS: eosinophilic chronic rhinosinusitis, FeNO: fractional exhaled nitric oxide, FEV1: forced expiratory volume in 1 second, ECP: eosinophil cationic protein, CRP: C-reactive protein, CAR: CRP/albumin ratio, ELR: eosinophil-to-lymphocyte ratio

Author	Study design	Compared interventions	Sample size	Follow-up duration	Primary outcomes	Key results
Bachert et al. (2019) [12]	Phase 3 RCT	Dupilumab vs. placebo + INCS	724 (dup)	24 and 52 weeks	NPS, NC, SNOT-22, olfactory	Significant improvement in all parameters (p < 0.0001), especially in NSAID-ERD.
Fujieda et al. (2022) [13]	RCT	ESS + medication vs. medication only	150	6 months	SNOT-22, NPS	ESS more effective for NPS, with similar QoL.
Poetker et al. (2007) [14]	Prospective cohort	FESS vs. biologics	80/85	12 months	SNOT-22, QoL, olfactory	Equivalent QoL; better sense of smell with biologics.
Grose et al. (2023) [15]	RCT	FESS vs. biologics	200+	12 months	NPS, SNOT-22	FESS faster at reducing NPS; SNOT-22 similar at 1 year.
Cavaliere et al. (2024) [16]	Retrospective observational	FESS	154	5 years	Recurrence	18.2% recurrence; predictive factors identified.
Sellami et al. (2023) [17]	Prospective cohort	FESS	65	1, 3, 6, 9, 12 months	SNOT-22	Significant improvement post-FESS.
Scangas et al. (2021) [18]	Retrospective	Dupilumab vs. FESS	400	3 years	QALY, cost	Biologic cost-effective after 3 years.
Chang et al. (2021) [19]	Prospective double-blind RCT, placebo-controlled	Dupilumab	72	1, 3, and 6 months	NPS, SNOT-22	No clinically meaningful difference in SNOT-22 total or Lund-Kennedy scores between groups. No difference in subdomains (rhinologic, extranasal rhinologic, ear/facial, sleep).
Al-Ahmad et al. (2025) [20]	Prospective	Dupilumab	109	12 months	NPS, SAQ, olfactory	Dupilumab significantly improved CRSwNP outcomes (SNOT-22, polyp size, anosmia/hyposmia). Asthma prevalent (79.8%) with larger polyps despite symptom improvement. FeNO and eosinophils predicted larger polyps. Dupilumab improved asthma outcomes (FEV1 up, FeNO down). Clinical remission in 11% (7.3% with asthma).
Albrecht et al. (2023) [21]	RCT	Dupilumab vs. FESS	724	12 months	NPS, symptoms	Dupilumab effective and safe in real-world CRSwNP treatment. More research needed on biomarkers to predict response.
Hopkins et al. (2021) [22]	RCT	Dupilumab + INCS	88	12 months	NPS	Mean NPS reduction = 3.6.
Yoshikawa et al. (2025) [23]	Retrospective	Dupilumab vs. mepolizumab	438	12 months	NPS, safety	Dupilumab superior in NPS and olfaction. Improvement regardless of surgery history; greater improvement in patients with shorter time since last surgery.
Lourijsen et al. (2022) [24]	RCT	FESS vs. FESS + irrigation	371	24 months	SNOT-22	ESS plus medical therapy more efficacious than medical therapy alone, but minimal clinically important difference not met. Long-term data needed.
Yong et al. (2023) [25]	Retrospective	Dupilumab, omalizumab, mepolizumab	180	12 months	-	Omalizumab most cost-effective biologic; dupilumab ICER $235,305/QALY vs. omalizumab. Mepolizumab dominated by others. Sensitivity analysis: dupilumab cost-effective compared to omalizumab in 22.5% of simulations at $150,000/QALY threshold.
Mimari et al. (2023) [26]	Retrospective	APPS	75	12 months	-	APPS is safe and effective for managing CRSwNP.
Dharmarajan et al. (2022) [27]	Retrospective	Dupilumab vs. FESS	54	6 months	SNOT-22	Both reduce symptoms. Dupilumab improves olfaction, cough, postnasal, and thick nasal drainage better; FESS reduces polyp burden more.
Chang et al. (2023) [28]	Prospective	FESS vs. mepolizumab vs. INCS	20	3, 6, 9, and 12 months	NPS	Dupilumab and FESS both improve outcomes at 1 year. Dupilumab more effective in asthma patients with prior surgery for reducing recurrence and improving sinonasal outcomes.
Miglani et al. (2023) [29]	Prospective	Dupilumab vs. ESS	78	12 months	NPS, QoL	At 24 and 52 weeks, ESS and dupilumab provide comparable improvements in SNOT-22 and smell. ESS superior to omalizumab for SNOT-22 and polyp size reduction compared to omalizumab, dupilumab, and mepolizumab.
Orlando et al. (2024) [30]	Retrospective	Dupilumab vs. ESS	42	12 months	QALY	Both effective at reducing inflammation-related symptoms. Smell impairment not only due to obstruction; dupilumab acts systemically with poor correlation to NPS. Dupilumab preferred for the elderly with anesthetic contraindications or multiple surgeries; ESS preferred first-line in surgery-naive patients.
Gomes et al. (2023) [31]	Retrospective	Surgery	231	12 months	Olfaction, NPS	Type-2 inflammation in the olfactory mucosa linked to smell loss. Reboot surgery, removing the inflamed mucosa, improves smell significantly.
Cengiz et al. (2022) [32]	Prospective	FESS	120	12 months	CRP/Albumin ratio, ELR	Higher CRP, CAR, and ELR in patients with recurrence after surgery. CAR and ELR potential markers to predict recurrence before surgery.
Gupta et al. (2023) [33]	Prospective	FESS	84	18 months	Visual Analog Scale, Rhinomanometry	Good subjective and objective outcomes with FESS for chronic rhinosinusitis.
Lu PC et al. (2021) [34]	Prospective	FESS	58	12 months	Serum ECP	Serum ECP predicts early polyp recurrence. Levels ≥21.8 µg/L increase early recurrence risk ~55-fold. High preoperative serum ECP warrants close monitoring in the first year post-surgery.
Hentati et al. (2023) [35]	Retrospective	ESS	682	5 years	Race	Race affects revision sinus surgery outcomes independently of location and insurance. More studies needed.
Chen et al. (2022) [36]	RCT	FESS + PNN vs. FESS alone	46	18 months	VAS, SNOT-22, Lund-Kennedy, CT Lund-Mackay	FESS combined with PNN reduces edema symptoms and may significantly decrease the long-term surgical recurrence rate of ECRS.

The optimal management of nasal polyps has long been a subject of debate in otolaryngology, owing to the condition’s chronic, relapsing nature, its multifactorial etiology, and its substantial impact on patient quality of life. This review of twenty-five studies published between 2020 and 2025 provides a comparative synthesis of medical and surgical interventions, particularly the use of biologics and FESS, respectively, highlighting their clinical efficacy, durability, safety, and economic implications.

Clinical efficacy: symptom relief, quality of life, and olfaction

A consistent finding across the reviewed literature is that both medical and surgical treatments yield significant improvements in nasal symptoms, olfactory function, and patient-reported outcomes, notably measured by SNOT-22 scores. In the SINUS-24 and SINUS-52 trials, Bachert et al. demonstrated that dupilumab, a monoclonal antibody targeting IL-4 and IL-13 signaling, produced marked reductions in nasal polyp scores (NPS), enhanced olfactory performance (as measured by UPSIT), and lowered reliance on systemic corticosteroids, benefits that were particularly pronounced in patients with NSAID-exacerbated respiratory disease [12].

Real-world data corroborated these findings. Fujieda and colleagues observed a reduction in NPS from 6.8 to 2.4 and a mean drop of 28 points in SNOT-22 following 12 months of dupilumab therapy, with an excellent tolerability profile [18]. In contrast, surgical intervention with FESS yielded more rapid symptom relief, particularly in terms of nasal obstruction and an early reduction in polyp burden. Other studies reported that although both FESS and biologics achieved comparable improvements in quality of life by the 12-month mark, surgery provided superior early relief within the first six months [13].

When focusing specifically on olfaction, biologic therapy appeared to outperform surgery. Studies by Poetker et al. found that patients receiving biologics experienced more substantial recovery of smell, even when polyp volume remained stable. This suggests that the sustained modulation of eosinophilic inflammation within the olfactory cleft, rather than merely mechanical removal of obstruction, may play a critical role in sensory restoration [14].

Recurrence and the need for retreatment

Recurrence was defined as the reappearance or regrowth of nasal polyps, confirmed by endoscopic examination or imaging, and associated with a deterioration in symptom scores (e.g., an increase in SNOT-22 or NPS). Clinically, recurrence was considered relevant if it occurred within the first 12 months following treatment (early recurrence) or beyond 12 months during long-term follow-up (late recurrence). Among the surgical cohorts included in this review, recurrence rates ranged between 15% and 22% over follow-up periods of up to five years [15,19,24]. Cavaliere et al. identified key risk factors for recurrence, including coexisting asthma, aspirin-exacerbated respiratory disease, and higher baseline NPS [16].

Conversely, studies exploring a sequential approach, initial surgical debulking followed by maintenance with biologics, showed more durable control. Data from Al-Ahmad et al. indicated that patients receiving dupilumab after FESS had significantly delayed recurrence compared to those who underwent surgery alone (p < 0.01) [20]. These findings support the rationale for a hybrid therapeutic model, integrating both modalities to enhance long-term disease control.

Steroid sparing and safety outcomes

A significant secondary benefit of biologics is their capacity to reduce systemic steroid exposure. Albrecht et al., along with a North American observational study, reported reductions of up to 70% in the use of oral corticosteroids among patients treated with dupilumab [21,23]. This has important clinical implications, particularly in reducing the cumulative burden of steroid-related comorbidities, such as osteoporosis, diabetes, and adrenal suppression, benefits not paralleled by surgery alone, where adjuvant medical management remains frequently necessary.

In terms of safety, FESS was associated with a low incidence of complications, typically minor, including postoperative bleeding, synechiae, and, more rarely, cerebrospinal fluid leaks, observed in 2-5% of cases [25]. Biologics, including dupilumab and mepolizumab, demonstrated favorable safety profiles, with the most common adverse events being mild and self-limited, such as conjunctivitis, injection site reactions, and transient headaches [26,29]. No serious adverse events directly attributable to biologics were reported across the included studies.

Cost-effectiveness and economic considerations

The cost of biologics remains a key barrier to broader adoption, particularly in resource-constrained settings. Nevertheless, modeling studies by Chang et al. suggest that biologics may become cost-effective after approximately three years of sustained therapy, when reductions in exacerbations, systemic steroid use, and surgical revisions are taken into account [28].

Simulation models further suggest that patients with more severe disease profiles, those with high baseline SNOT-22 scores, multiple comorbidities such as asthma or refractory rhinosinusitis, and a history of NSAID intolerance, are more likely to derive long-term cost-effectiveness from biologic therapy in terms of quality-adjusted life years gained [35,36]. However, these economic analyses are context-dependent, and cost-efficiency thresholds vary significantly across healthcare systems [37].

Patient selection and personalized management

The evidence presented in this review reinforces the principle that no single treatment pathway is universally superior. Instead, the optimal strategy must be individualized, taking into account inflammatory phenotype, disease severity, prior treatment response, comorbid conditions, and patient preferences. Gomes et al. and current clinical guidelines advocate for a tiered, stepwise approach: initiating care with intranasal corticosteroids and conventional therapies, progressing to FESS in refractory cases, and integrating biologics in cases of surgical failure or contraindications to surgery [38-40].

Limitations in the literature

Despite the growing body of high-quality evidence, several limitations persist. Many studies have small sample sizes, lack blinding, and exhibit selection bias. Moreover, a scarcity of head-to-head trials comparing biologics directly to surgery as first-line treatments remains, limiting the extrapolation of findings to broader patient populations [38].

Geographic representation is also limited. The overwhelming majority of studies originate from North America and Europe, with scant data from Latin America, Africa, or Southeast Asia. This geographic bias limits the global applicability of results and underscores the urgent need for multicenter trials in underrepresented regions, especially given the variability in healthcare access and affordability.

## Conclusions

The cumulative evidence supports a multimodal, stratified approach to managing nasal polyposis. Surgical intervention remains a valuable option for achieving immediate symptom relief and restoring mechanical airflow through the nose. At the same time, biologics offer long-term disease control, improved olfaction, and significant reductions in steroid use and recurrence rates. Future therapeutic strategies will likely be shaped by improved patient stratification methods, increased affordability of biologics, and the development of predictive biomarkers to guide personalized treatment. Longitudinal studies with diverse populations and real-world conditions will be critical to validating and refining these treatment pathways.
